# Heat Pre-Treatment Modified Host and Non-Host Interactions of Powdery Mildew with Barley Brassinosteroid Mutants and Wild Types

**DOI:** 10.3390/life14010160

**Published:** 2024-01-22

**Authors:** Magdalena Rys, Diana Saja-Garbarz, József Fodor, Jakub Oliwa, Gábor Gullner, Csilla Juhász, Andrzej Kornaś, Andrzej Skoczowski, Damian Gruszka, Anna Janeczko, Balázs Barna

**Affiliations:** 1 Polish Academy of Sciences, The Franciszek Górski Institute of Plant Physiology, Niezapominajek 21, 30-239 Krakow, Poland; 2 Plant Protection Institute, Centre for Agricultural Research, HUN-REN, Herman Ottó út 15, 1022 Budapest, Hungary; 3 Institute of Biology and Earth Sciences, University of the National Education Commission, Krakow, Podchorążych 2, 31-054 Krakow, Poland; 4 Institute of Biology, Biotechnology and Environmental Protection, Faculty of Natural Sciences, University of Silesia, Jagiellonska 28, 40-032 Katowice, Poland

**Keywords:** *Blumeria hordei*, *Blumeria graminis*, chlorophyll *a* fluorescence, leaf spectral reflectance, near-isogenic lines, pathogen inoculation

## Abstract

High temperatures associated with climate change may increase the severity of plant diseases. This study investigated the effect of heat shock treatment on host and non-host barley powdery mildew interactions using brassinosteroid (BR) mutants of barley. Brassinosteroids are plant steroid hormones, but so far little is known about their role in plant-fungal interactions. Wild type barley cultivar Bowman and its near-isogenic lines with disturbances in BR biosynthesis or signalling showed high compatibility to barley powdery mildew race A6, while cultivar Delisa and its BR-deficient mutants 522DK and 527DK were fully incompatible with this pathogen (host plant-pathogen interactions). On the other hand, Bowman and its mutants were highly resistant to wheat powdery mildew, representing non-host plant-pathogen interactions. Heat pre-treatment induced shifts in these plant-pathogen interactions towards higher susceptibility. In agreement with the more severe disease symptoms, light microscopy showed a decrease in papillae formation and hypersensitive response, characteristic of incompatible interactions, when heat pre-treatment was applied. Mutant 527DK, but not 522DK, maintained high resistance to barley powdery mildew race A6 despite heat pre-treatment. By 10 days after heat treatment and infection, a noticeable shift became apparent in the chlorophyll *a* fluorescence and in various leaf reflectance parameters at all genotypes.

## 1. Introduction

Climate change signifies long-term shifts in temperatures and weather patterns. Regardless of the causes (natural changes in the sun’s activity, large volcanic eruptions, human industrial activity), climate changes are connected to global warming, which denotes an overall rise in global temperatures [1,2,3]. In Poland, the last decade was exceptionally warm compared to previous periods [4]. The average annual temperature reached as much as 9.1 °C. By comparison, the average in the reference period of 1961–1990 was 7.5 °C. There was an exceptional increase in the number of hot days, with the maximum temperature exceeding 30 °C. The impact of a warming climate on fungal diseases in plants has been emphasized by Stukenbrock and Gurr [5]. They highlighted that “little is known about the response of major plant pathogens to climate change, increasing temperatures in the Northern Hemisphere will drive the evolution of new temperature tolerances in fungal pathogens, and the establishment of pathogens that previously were restricted to more southerly regions”. This issue was also previously reviewed by Yáñez-López et al. [6]. In the UK, the effects of climate change on Phoma were assessed, revealing that epidemics will not only increase in severity but also spread northwards [7]. As for downy mildew (*Plasmopara viticola*) on grapevine, there is a predicted increase in disease pressure with each decade due to more favourable temperature conditions [8].

Barley is one of the most important cereals grown in the world [9], but apart from adverse meteorological and soil conditions, substantial yield losses in this crop are caused by fungal diseases [10]. One such disease is powdery mildew, caused by a biotrophic fungal pathogen *Blumeria hordei* (*Bh*), that can be efficiently controlled by inexpensive and environmentally-friendly genetic resistance [11,12]. However, it is important to note that changing climate (connected to elevated temperatures) may promote the breaking down of this resistance. Heat pre-treatment modified host and non-host interactions [13], and infection of barley by *Bh,* results in a so-called host plant-pathogen interaction, but barley in specific circumstances (under high temperature stress) may be attacked also by *Blumeria graminis* f. sp. *tritici* (*Bgt*; powdery mildew of wheat), representing a non-host interaction. The mechanisms behind this phenomenon are only partly understood [14,15,16,17]. Although non-host resistance is considered as the most durable and efficient immune system of plants, and has been investigated at multiple levels of plant defence for several decades, there are still many open questions regarding the underlying mechanisms [18,19,20,21].

Several studies have shown that heat stress usually negatively affects key plant disease resistance mechanisms [22,23]. Heat shock (a short-term exposure to high temperatures), e.g., submerging plants in 49 °C water for 20 s (s) one day before *Bh* inoculation, resulted in the susceptibility of near-isogenic lines of barley (*H. vulgare* cv. Ingrid) containing different resistance genes (*mlo5*, *Mlg*, *Mla12*). The same heat shock further increased susceptibility to *Bh* in genetically susceptible barley (cv. Ingrid Mlo) [24].

Numerous regulators are involved in plant-pathogen interactions. The role of plant steroid hormones–brassinosteroids (BR) remains relatively underexplored. BR belong to polyhydroxylated sterol derivatives discovered in the late 1970s [25,26,27]. BR control development of plants but also participate in regulation of plant responses to abiotic and biotic stresses [28,29]. BR treatment improved plant resistance to viral, bacterial and fungal infections [30,31,32,33]. However, not much is known about the effects of lowered contents of endogenous BR or the effects of disturbances in BR signalling on the resistance of plants to fungal pathogens. The pathogen resistance of the barley *uzu* mutant (the first semi-dwarf mutant), with disturbances in the BR signalling, has been studied by Ali et al. [30]. The mutation is located within the *HvBRI1* (*Uzu1*) gene encoding a transmembrane BR receptor [34]. Ali et al. [30] also performed pathogenicity tests using the obligate pathogen Barley stripe mosaic virus, the necrotrophic net blotch pathogen *Pyrenophora teres*, and the toxigenic hemibiotrophic fungus *Fusarium culmorum* causing Fusarium head blight. Surprisingly, BR mutants were more resistant to all three pathogens and showed significantly attenuated symptoms as compared to wild type plants. Further, in barley, the disruption of BRI1 improved the disease resistance against necrotrophic and hemibiotrophic pathogens, but had no effect on biotrophic pathogens [35]. Our earlier studies [36] showed that the mutation in *HvDWARF* (in barley genotype 527DK) did not affect resistance to powdery mildew (no appearance of symptoms). Interestingly, alterations in hormonal homeostasis were observed between uninoculated and inoculated plants of mutant and wild type. Inoculation with the pathogen markedly induced the accumulation of compounds from auxin group (indole-3-carboxylic acid, oxindole-3-acetic acid, 4-chloroindole-3-acetic acid, and 5-chloroindole-3-acetic acid). The effect was more pronounced in mutant 527DK than in wild type plants. However, there is still limited knowledge regarding the role of BR in the response of plants to fungal pathogens–and particularly the effect of various environmental stressors (abiotic stresses) on pathogen resistance of plants with disturbances of biosynthesis of BR or BR signalling. Considering that climate change is associated with increased temperatures, drought, and appearance of invasive plant pathogens, the present research aimed to investigate the effect of high temperature on sensitivity of barley to powdery mildew in both host and non-host interactions. In the experiments, we focused also on answering the question of whether BR deficiency or signalling disturbances in near isogenic lines (NILs) of Bowman and in mutants of Delisa modify heat-induced sensitivity of barley to these host and non-host fungal pathogens. Therefore, for the main experiment and more detailed research, we selected two representative lines: BW084 (BR-deficient) and BW312 (BR-insensitive). On the other hand, we showed that the barley cultivar Delisa and its semi-dwarf BR-deficient mutants 522DK and 527DK showed high resistance to this particular powdery mildew race [36]. Since we aimed to compare changes between compatible and incompatible interactions in these two barley cultivars (Bowman and Delisa) and their BR mutants, the non-host pathogen wheat powdery mildew was also used for inoculation of Bowman plants. Since barley plants normally display symptomless resistance to wheat powdery mildew, a short heat pre-treatment was applied to partially suppress the level of resistance of Bowman barley and NILs to *Bgt*. Similarly, for Delisa and its mutants, barley powdery mildew (incompatible interaction) was used for inoculation with or without previous heat pre-treatment which made the plants more susceptible.

## 2. Materials and Methods

### 2.1. Plant Material, Pathogen Inoculation, and Heat Pre-Treatment

Plant materials in these studies included the semi-dwarf barley near-isogenic lines (NILs) BW084 (BR-deficient) and BW312 (with BR signalling disturbances [BR-insensitive]) with the reference cultivar Bowman, and the barley cultivar Delisa (wild type) with its semi-dwarf BR-deficient mutants 522DK and 527DK. Seeds were obtained from the collection of the Department of Genetics of the University of Silesia, Poland. A detailed description of the plant material can be found in Dockter et al. [37], Gruszka et al. [38] and Gruszka et al. [39].

Barley seeds were sown in Petri dishes containing water-soaked filter paper and incubated for three days at 24 °C in darkness. Subsequently, germinated seeds were transferred to 10 cm diameter pots filled with soil (seven seeds per pot) and grown under greenhouse conditions with natural light in May and a temperature range of 23 ± 2 °C.

In order to investigate the host−pathogen interactions, all barley genotypes (Bowman, Delisa, BW084, BW312, 522DK, and 527DK) were inoculated with the fungal pathogen barley powdery mildew, *Blumeria hordei* (*Bh*) race A6, kindly supplied by Karl-Heinz Kogel (Justus Liebig University, Giessen, Germany). Barley powdery mildew was maintained on susceptible host plants (cv. Ingrid Mlo) in growth chambers (20 °C, 60% relative humidity, 16 h photoperiod with a light intensity of 100 μmol m^−2^ s^−1^). For inoculation, conidia from heavily infected barley plants were dusted equally onto the primary leaves of 8-day-old barley seedlings. The inoculum density averaged 50 conidia mm^−2^. 

Furthermore, 8-day-old seedlings of Bowman and its NILs (BW084, BW312) were inoculated with the non-host pathogen wheat powdery mildew fungus (*Blumeria graminis* f. sp. *tritici*). The wheat powdery mildew was collected in the greenhouse of the Plant Protection Institute, Centre for Agricultural Research, HUN-REN (Hungary), and maintained on a susceptible wheat cultivar Alcedo under the same growth chamber conditions as described above. 

Heat shock treatment of barley leaves was carried out according to Barna et al. [24]. Leaves of 7-day-old barley plants were immersed in 49 °C water for 30 s 24 h before inoculation with wheat or barley powdery mildew. Visual evaluation of disease symptoms was carried out continuously.

### 2.2. Measurements and Observations

#### 2.2.1. Localization of Hydrogen Peroxide Accumulation and Microscopy

Detection of H_2_O_2_ was performed by an endogenous peroxidase-dependent in situ histochemical staining using 3,3′-diaminobenzidine tetrahydrochloride (DAB) as described by Thordal-Christensen et al. [40]. Detached leaves were vacuum infiltrated with 1 mg mL^−1^ DAB dissolved in water and incubated at room temperature for 4 h before fixation.

Plant−pathogen interactions were evaluated under the microscope. For microscopy, primary leaves were harvested at 2, 3, 5, and 7 days after inoculation. Leaf fixation, bright-field and fluorescence microscopy were performed as described by Hückelhoven and Kogel [41]. Excised leaves were transferred to 0.15% (*w*/*v*) trichloroacetic acid in ethanol: chloroform 4:1 and stored in 50% glycerol prior to staining and microscopy. To stain fungal structures for bright-field microscopy, leaves were incubated in 10% blue ink (Pelikan) in 25% acetic acid for 1 min and then washed with water to remove the excess ink. Whole-cell and papilla autofluorescence was observed by fluorescence microscopy using a fluorescence microscope (Olympus BX51, Tokyo, Japan) equipped with a 480 nm excitation filter, a 500 nm dichroic mirror and a 515 nm barrier filter. Interaction sites were evaluated for conidial germination, fungal developmental stages, papilla formation, accumulation of autofluorescent phenolic compounds, hypersensitive reaction (HR), and H_2_O_2_ accumulation. Four leaves, each representing at least 100 plant/fungus interaction sites, were scored for each genotype and experiment.

#### 2.2.2. Fast Kinetics of Chlorophyll *a* Fluorescence

Measurements of chlorophyll *a* fluorescence were performed 3 and 10 days after inoculation (DPI) in Bowman and its NILs inoculated by *Bgt,* and in Delisa and one mutant (527DK) inoculated by *Bh*. Chlorophyll *a* fluorescence kinetics parameters were detected using a Handy-PEA fluorimeter (Plant Efficiency Analyser [PEA], Hansatech Ltd., Pentney, UK) to estimate the efficiency of photosystem II (PSII). Measurements were taken in the central parts of second leaves adapted to the dark for 30 min using special clips. The measurements were performed on 15 leaves (*n* = 15 repetitions) from each genotype and treatment. The kinetics of chlorophyll *a* fluorescence were described by a fluorescence induction curve known as OJIP-test. 

Chlorophyll *a* fluorescence induction curves (read using PEA) were analyzed and the following steps were used to perform the JIP test: O-20 μs, J-200 μs, I-30 ms, P-300 ms. Difference curves of fluorescence kinetics were calculated by subtracting the normalized (to points O and P) fluorescence values of treated plants from the normalized control values, separate for each genotype, according to the methodology of Oukarroum et al. [42].

The following fluorescence parameters were calculated based on fluorescence curves: the maximum quantum yield of the photosystem II primary photochemistry (Fv/Fm ratio), the maximum quantum yield of primary photochemistry (Fv/F_0_), φ_(Eo)_, as well as the PSII performance index (PI abs). Moreover, the following parameters of phenomenological energy fluxes were calculated from the fluorescence curve: the energy absorption by the antenna pigments (ABS/CSm), the amount of energy that was trapped in the reaction centre (TRo/CSm), the energy flux for the electron transport (ETo/CSm), and the dissipation of energy as heat (DIo/CSm) where CS is the sample cross-section. The same parameters were also calculated for the reaction centre (RC). Detailed equations for the specific parameters are given in Strasser et al. [43].

#### 2.2.3. Analysis of Leaf Spectral Properties (Reflectance)

The reflectance spectrum of light radiation in the range 400–1000 nm was measured using the CID Bio-Science CI-710s spectrometer (CID Bio-Science, Camas, WA, USA). Measurements were taken in Bowman and its NILs inoculated by *Bgt*, and in Delisa and one mutant (527DK) inoculated by *Bh* 3 and 10 DPI. The reflectance was measured on the upper (adaxial) surface of the second leaf. For each genotype, the measurement was performed on 15–30 leaves. Based on the reflectance spectra, the following reflection parameters were calculated: Carotenoid Reflectance Index (CRI1) [44]; Structure-Insensitive Pigment Index (SIPI), indicating carotenoid/chlorophyll a ratio [45]; Water Band Index (WBI), indicating plant water status [46], and Photochemical Reflectance Index (PRI), which is a simplified indicator of photosynthetic efficiency [47].

## 3. Results

### 3.1. Effect of Powdery Mildew and Heat Pre-Treatment on Disease Symptoms

**Figure 1:**
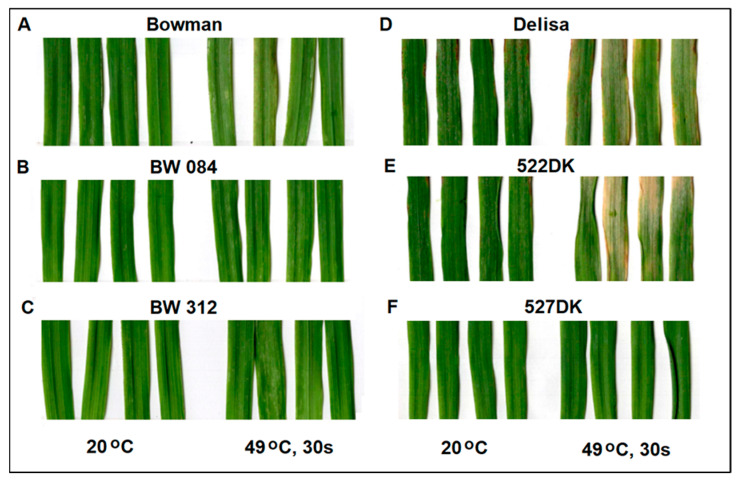
Macroscopic symptoms on primary leaves of barley plants seven days after inoculation with powdery mildew. Barley plants of Bowman (**A**) and NILs (BW084 (**B**), BW312 (**C**)) were inoculated with wheat powdery mildew (*Blumeria graminis* f. sp. *tritici*). Delisa (**D**) (and mutants 522DK (**E**), 527DK (**F**)) were inoculated with barley powdery mildew (*Blumeria hordei*). Inoculation was performed on 8-day-old seedlings 24 h after heat treatment (49 °C water for 30 s). Heat-untreated plants were maintained at 20 °C throughout the experiment. In photographs (**A**–**F**) for each genotype four leaves on the left side are from control (growing at 20 °C) while four leaves on the right side are from heat pre-treated plants. Photographs are from one representative experiment; three inoculation experiments were carried out with similar results.

As our earlier studies revealed, cultivar Bowman and NILs BW084 and BW312 (and also some other tested genotypes, such as BW091, BW333 and BW885) were all highly susceptible to *Bh* race A6, while the barley cultivar Delisa and its semi-dwarf BR-deficient mutants 522DK and 527DK proved to be highly resistant to this barley powdery mildew race (Figure 1D–F).

As expected, Bowman and NILs were highly resistant to wheat powdery mildew (*Bgt*), no visible symptoms could be detected (Figure 1A–C). However, heat treatment 24 h before powdery mildew infection made the plants markedly more susceptible (Figure 1A–C). 

**Figure 2:**
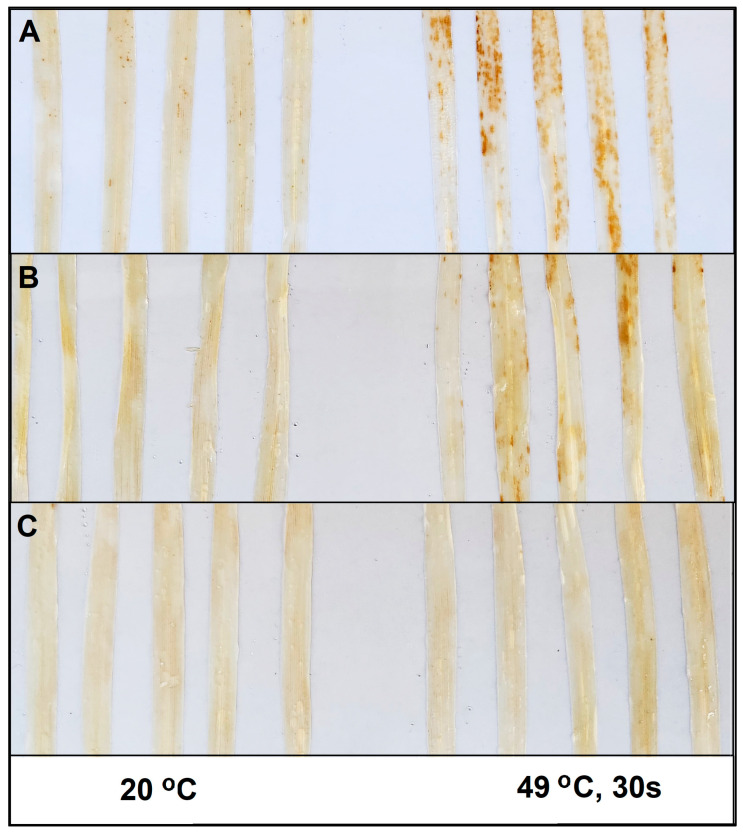
Hydrogen peroxide accumulation detected by 3, 3′-diaminobenzidine (DAB) in primary leaves of the barley cultivar Delisa (**A**), 522DK (**B**) and 527DK (**C**) seven days after inoculation with *Blumeria hordei* race A6. Control plants were kept at 20 °C (leaves on left side), heat pre-treated plants (leaves on right side) were immersed into 49 °C water for 30 s 24 h before inoculation. Photographs are from one representative experiment; the experiment was repeated three times with similar results.

In addition, due to high-temperature pre-treatment, the highly resistant Delisa and the BR-mutant 522DK became infected with *Bh* race A6, showing remarkable sporulation (Figure 1D,E). Heat pre-treatment also resulted in a development of macroscopical necrotic lesions at late stages of infection, which was accompanied by an H_2_O_2_ accumulation as indicated by DAB staining (Figure 2A,B). Interestingly, very mild visible symptoms of powdery mildew (*Bh*) infection were detectable on leaves of mutant 527DK even after heat pre-treatment (Figure 1F), which was confirmed also by DAB staining (Figure 2C).

### 3.2. Microscopy and Localization of Hydrogen Peroxide Accumulation

**Figure 3:**
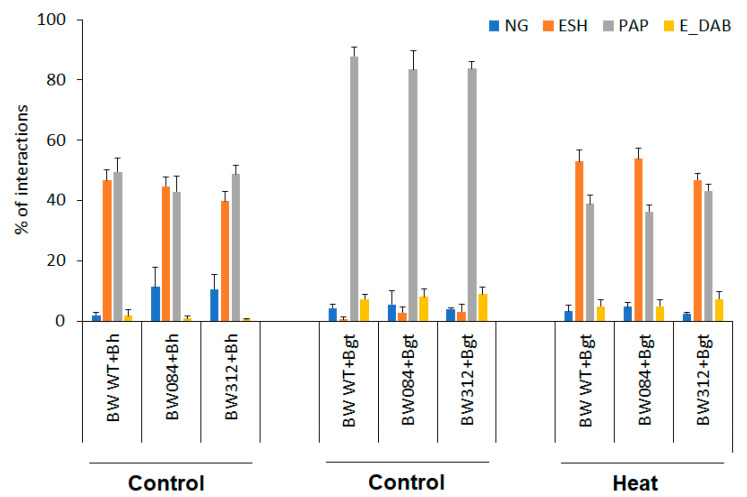
Microscopic investigations of interactions of Bowman and NILs (BW 084 and BW312) with barley powdery mildew (*Bh*) race A6 or wheat powdery mildew (*Bgt*) 2 days after inoculation. Heat pre-treatment was applied 24 h before inoculation, control plants were kept at 20 °C. Types of plant−pathogen interactions: NG—non-germinated conidia, ESH—elongated secondary hyphae (compatible interaction), PAP—formation of papillae (incompatible interaction), E-DAB—epidermal cells with whole-cell DAB staining (putative epidermal HR). Five leaves, each representing at least 100 plant/fungus interaction sites, were scored for each genotype and experiment. Data represent mean values ± SD.

When the interactions between barley powdery mildew and Bowman with its NILs (BW084 and BW312) were investigated microscopically, the number of non-germinated conidia, elongated secondary hyphae, papilla formation, and DAB-stained epidermal cells (indicating epidermal HR) were counted (Figure 3).

In the case of barley powdery mildew-inoculated Bowman plants, the high frequency of fungal penetration and development of elongated secondary hyphae indicated compatibility. Interestingly, the rate of papillae formation was also high (Figure 3). There was one significant difference between Bowman and the NILs: the number of non-germinated conidia significantly increased on both NILs, indicating a certain type of elevation of resistance (Figure 3).

**Figure 4:**
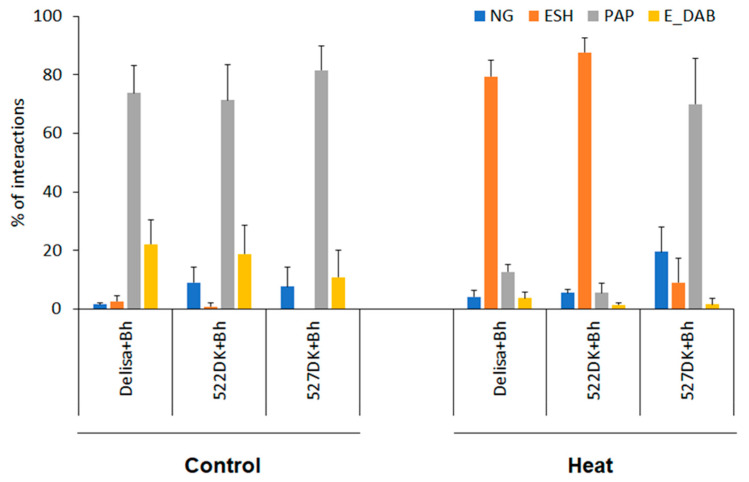
Microscopic investigations of interactions of Delisa and its mutants with *Bh* two days after inoculation. Heat pre-treatment was applied 24 h before inoculation, control plants were kept at 20 °C. Types of plant−pathogen interactions: NG—non-germinated conidia, ESH—elongated secondary hyphae (compatible interaction), PAP—formation of papillae (incompatible interaction), E-DAB—epidermal cells with whole-cell DAB staining (putative epidermal HR). Five leaves, each representing at least 100 plant/fungus interaction sites, were scored for each genotype and experiment. Data represent mean values ± SD.

The host incompatible interactions between Delisa or its mutants with *Bh* race A6 showed similar results as Bowman or its mutants with *Bgt* in non-host interactions, except that the rate of papillae formation and the number of epidermal HR were higher (Figure 3 and Figure 4). Interestingly, the number of non-germinated conidia was higher on 522DK than on Delisa, but 527DK did not show significant difference in this parameter. 

Heat pre-treatment induced disease susceptibility in Delisa and 522DK, but much less in 527DK. This could be seen from the high number of elongated secondary hyphae and low number of papillae formation on heat pre-treated Delisa and 522DK, but low number of elongated secondary hyphae and high number of papillae on 527DK (Figure 4).

**Figure 5:**
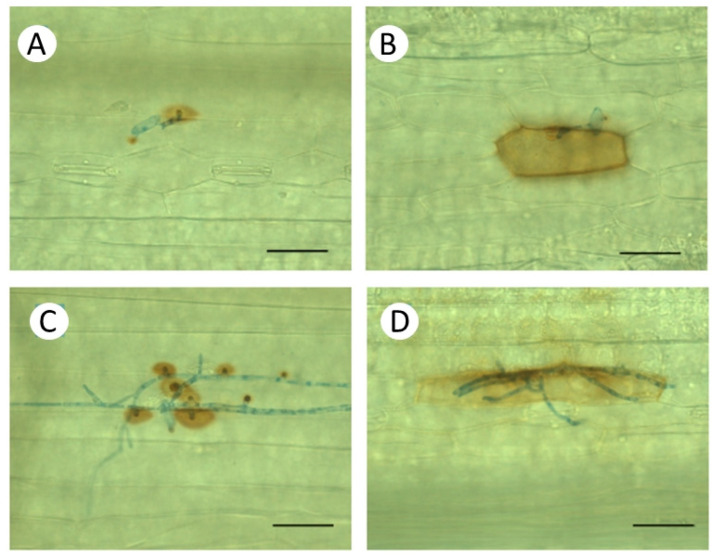
Non-host interaction of primary leaves of Bowman barley plants with wheat powdery mildew (*Blumeria graminis* f. sp. *tritici, Bgt*) three days after inoculation. Accumulation of H_2_O_2_ was detected by 3, 3′-diaminobenzidine (DAB). Upper panels (**A**,**B**), Control plants kept at 20 °C. Lower panels (**C**,**D**), Heat pre-treated plants were immersed into 49 °C water for 30 s 24 h before inoculation. (**A**,**C**) Papilla formation. (**C**,**D**) Whole-cell DAB staining in the epidermal cell attacked by *Bgt*, indicating an ongoing hypersensitive response (HR) leading to cell death. Bar: 50 μm.

The non-host interaction between Bowman and the NILs with *Bgt* showed extremely high rates of papillae formation and significantly higher frequency of epidermal HR than the compatible interaction with *Bh* (Figure 3). Due to the incompatibility, the rate of formation of elongated secondary hyphae was very low, practically zero. When susceptibility was induced by heat shock pre-treatment in the same barley genotypes, formation of elongated secondary hyphae of *Bgt* was even higher, and papillae formation was lower than in the original compatible interaction with *Bh* (Figure 3). It is to be noted, however, that in spite of an initial hyphal growth of *Bgt* on heat pre-treated plants, extensive papilla formation and epidermal HR were observed as defence responses in attacked epidermal cells (Figure 5C,D). As a result of the inhibition of fungal growth, the numbers of sporulating colonies remained low (Figure 1).

### 3.3. Chlorophyll a Fluorescence

**Figure 6:**
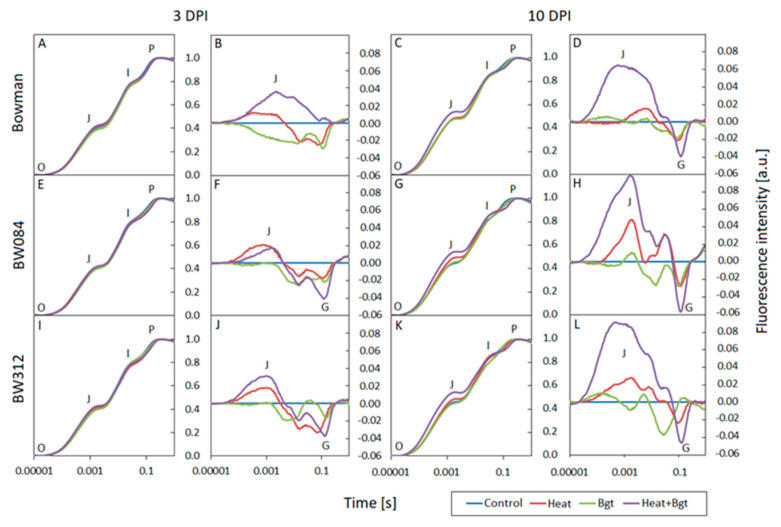
Normalized chlorophyll *a* fluorescence induction curves (OJIP) and difference curves calculated by subtracting the normalized (to points O and P) fluorescence values of treated plants from the control values for each genotype: Bowman (**A**–**D**), BW084 (**E**–**H**), BW312 (**I**–**L**), at 3 and 10 DPI by *Blumeria graminis* f. sp. *tritici*, *Bgt*. Heat pre-treated plants were immersed into 49 °C water for 30 s 24 h before inoculation.

**Figure 7:**
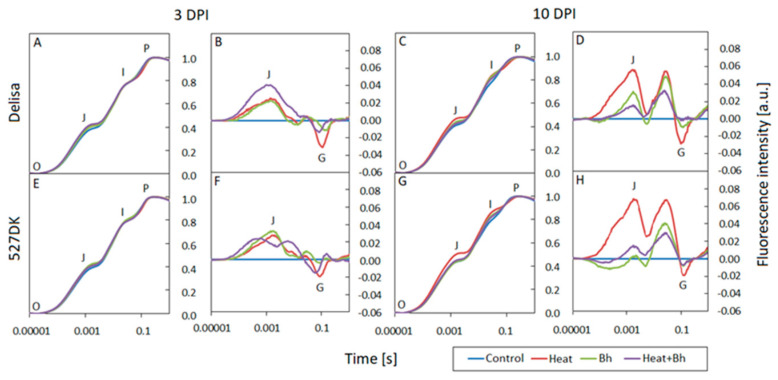
Normalized chlorophyll *a* fluorescence induction curves (OJIP) and difference curves calculated by subtracting the normalized (to points O and P) fluorescence values of treated plants from the control values for each genotype: Delisa (**A**–**D**), 527DK (**E**–**H**), at 3 and 10 DPI by *Blumeria hordei*, *Bh*. Heat pre-treated plants were immersed into 49 °C water for 30 s 24 h before inoculation.

At 3 DPI, the normalized fluorescence induction curves (OJIP) for the individual genotypes and treatments show no deviation from the controls (Figure 6A,E,I and Figure 7A,E). The curves have all the typical steps: O-J-I-P and follow a similar course. However, by 10 DPI, after heat treatment and infection, noticeable differences became apparent in all genotypes as compared to the control: there was a faster increase in fluorescence between steps O and J as well as a slower increase in fluorescence between steps I and P. This was observed at 10 DPI in Bowman Heat+*Bgt* (and NILs) and Delisa and 527DK plants at high temperature (Figure 6C,G,K and Figure 7C,G). The differences in the response of individual genotypes exposed to various treatments are more clearly visible on the difference curves in the form of an increase in fluorescence intensity in the J band, and a decrease in fluorescence in the G band (relative to the control, Figure 6B,F,J,D,H,L and Figure 7B,F,D,H). Importantly, changes in fluorescence intensity in the mentioned bands were noticeable already at 3 DPI, even before the macroscopic symptoms of infection were visible in the leaves.

**Figure 8:**
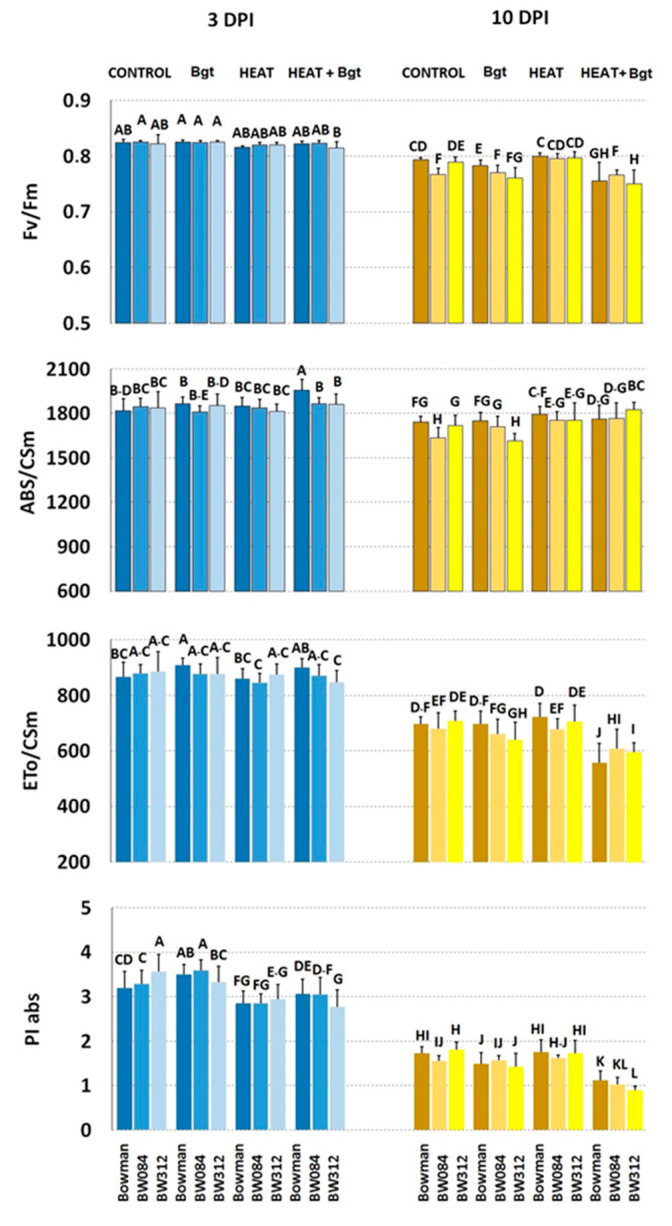
Selected parameters of chlorophyll *a* fluorescence characterizing PS II efficiency in plants of Bowman and its NILs (BW084, BW312) growing under control conditions (Control), inoculated by wheat powdery mildew (*Bgt*), exposed to heat (Heat), and heat pre-treated + inoculated by wheat powdery mildew (Heat+*Bgt*). Mean values (±SD) marked with the same letters did not differ significantly at *p* ≤ 0.05 according to Duncan’s test; DPI—days post inoculation. Heat pre-treated plants were immersed into 49 °C water for 30 s 24 h before inoculation. Measurements were carried out on 11 (3 DPI) and 18 (10 DPI) day old plants.

**Figure 9:**
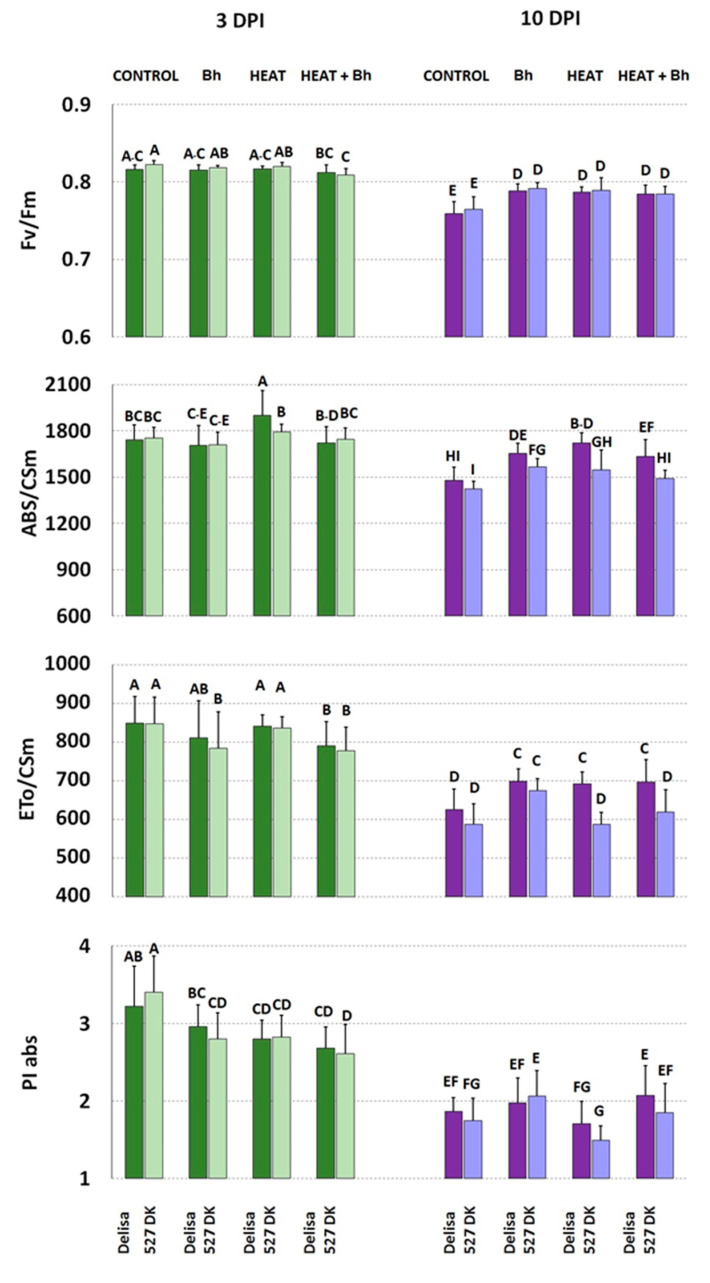
Selected parameters of chlorophyll *a* fluorescence characterizing PS II efficiency in plants of Delisa and its mutant 527DK growing under control conditions (Control), inoculated by barley powdery mildew (*Bh*), exposed to heat (Heat), and heat pre-treated + inoculated by barley powdery mildew (Heat+*Bh*). Mean values (±SD) marked with the same letters did not differ significantly at *p* ≤ 0.05 according to Duncan’s test; DPI—days post inoculation. Heat pre-treated plants were immersed into 49 °C water for 30 s 24 h before inoculation. Measurements were carried out on 11 (3 DPI) and 18 (10 DPI) day old plants.

Based on fluorescence curves, selected parameters were calculated and presented in Figure 8 and Figure 9 and Supplementary Tables S1 and S2.

At the earlier stage (3 DPI; Figure 8) no significant difference was observed in the measured parameters between uninoculated wild type Bowman and BR mutant NILs, except for PI abs which was higher in BW312. However, at a later stage (10 DPI) uninoculated BW084 showed significantly lower values of Fv/Fm and of ABS/CSm compared to the wild type and line BW312 (Figure 8). In response to the non-host interaction with *Bgt* at 3 DPI, ETo/CSm significantly increased in Bowman, and PI abs increased in Bowman and BW084, but decreased in BW312. Additionally, at 3 DPI, heat treatment significantly decreased PI abs in all barley lines as compared to their uninoculated controls. The combination of heat treatment and wheat powdery mildew infection, which induced susceptibility to a certain extent, resulted in an increase of ABS/CSm (but not ETo/CSm) in Bowman as compared to the uninoculated and untreated Bowman. In NILs, heat treatment and inoculation with *Bgt* did not significantly affect these parameters. In summary, Fv/Fm values were comparable across all genotypes and treatments. PI abs values were generally reduced after heat pre-treatment and heat pre-treatment + inoculation in all genotypes. Interestingly, at 3 DPI, infection alone in Bowman and BW084 caused an increase in PI abs (compared to control plans) while the effect was the opposite in BW312. 

In general, there were more pronounced changes in fluorescence parameters at a later stage (10 DPI). Primarily, there was a significant decrease in PI abs (alongside a decrease in Fv/Fm values) across all genotypes regardless of any treatment when comparing the results at 10 DPI and 3 DPI. 

By 10 DPI, wheat powdery mildew inoculation alone decreased Fv/Fm in Bowman and BW312 (but not in BW084), compared to the controls. This effect was further enhanced by the combination of heat pre-treatment and inoculation in Bowman and BW312 (but still not in BW084).

ABS/CSm decreased in BW312 (but increased in BW084) upon *Bgt* inoculation alone. In the case of Bowman, ABS/CSm did not change significantly in inoculated and control plants. ETo/CSm decreased only in inoculated BW312. 

Generally, among all the treatments, heat pre-treatment followed by pathogen inoculation caused the most substantial decrease in the values of PI abs and ETo/CSm parameters, which was accompanied relatively well by high values of ABS/CSm (characterizing heat-treated plants). Within this treatment, BW312 (but not BW084) exhibited a significantly lower value of PI abs than wild type Bowman. On the other hand, both heat pre-treated and inoculated NILs maintained higher values of ETo/CSm than the wild type Bowman. 

Among the other calculated fluorescence parameters presented in Supplementary Table S1, such as Fv/Fo, ABS/RC, DIo/RC, ETo/RC, φ_(Eo)_, DIo/CSm, TRo/CSm), particular attention should be paid to DIo/CSm and φ_(Eo)_. It is evident that energy loss as heat (expressed by DIo/CSm) increased in heat pre-treated and pathogen inoculated barley (especially in Bowman and BW312) already after 3 DPI, with this effect further enhanced by 10 DPI. Concerning the values of φ_(Eo)_, it is worth noting that in younger control plants there were no differences between Bowman and NILs, while in the case of older plants NILs maintained higher values of this parameter than Bowman. The trends were similar after inoculation at both 3 DPI and 10 DPI. Heat treatment generally deceased φ_(Eo)_ values compared to control plants, with a stronger effect observed at 10DPI. In heat pre-treated and inoculated plants the values of φ_(Eo)_ were further decreased. In this case, insignificant differences between Bowman and NILs were observed. 

In experiments involving host incompatible interactions between Delisa or 527DK and barley powdery mildew race A6 with or without heat pre-treatment, the following results were observed (Figure 9). At 3 DPI, an increase in ABS/CSm was only found in Delisa as a result of heat treatment. PI abs generally decreased in both Delisa and mutants at 3 DPI after all treatments, in comparison to untreated control. A decrease in ETo/CSm was found also in Delisa and 527DK after heat treatment and inoculation (elevated susceptibility). In addition, Fv/Fm decreased only in 527DK by combined heat treatment and inoculation. At a later stage (10 DPI) we detected a general decrease in all photosynthetic parameters in both barley genotypes. Interestingly, at 10 DPI all treatments in both genotypes increased Fv/Fm and ABS/CSm (except combined heat treatment and inoculation in 527DK). ETo/CSm was elevated in Delisa by all treatments. There was a similar though less pronounced increase in the values of this parameter in 527DK after all treatments. Among the parameters shown in Supplementary Table S2, it is worth noting the consistently reduced values of φ_(Eo)_ after all treatments at 3DPI; this trend was maintained at 10DPI. Energy loss as heat (DIo/CSm) showed a slight (statistically insignificant) tendency to increase in Delisa and mutants at 3 DPI after heat treatment, and in heat pre-treated + inoculated plants; however, this effect was not sustained at 10 DPI.

### 3.4. Leaf Spectral Properties (Leaf Reflectance)

**Figure 10:**
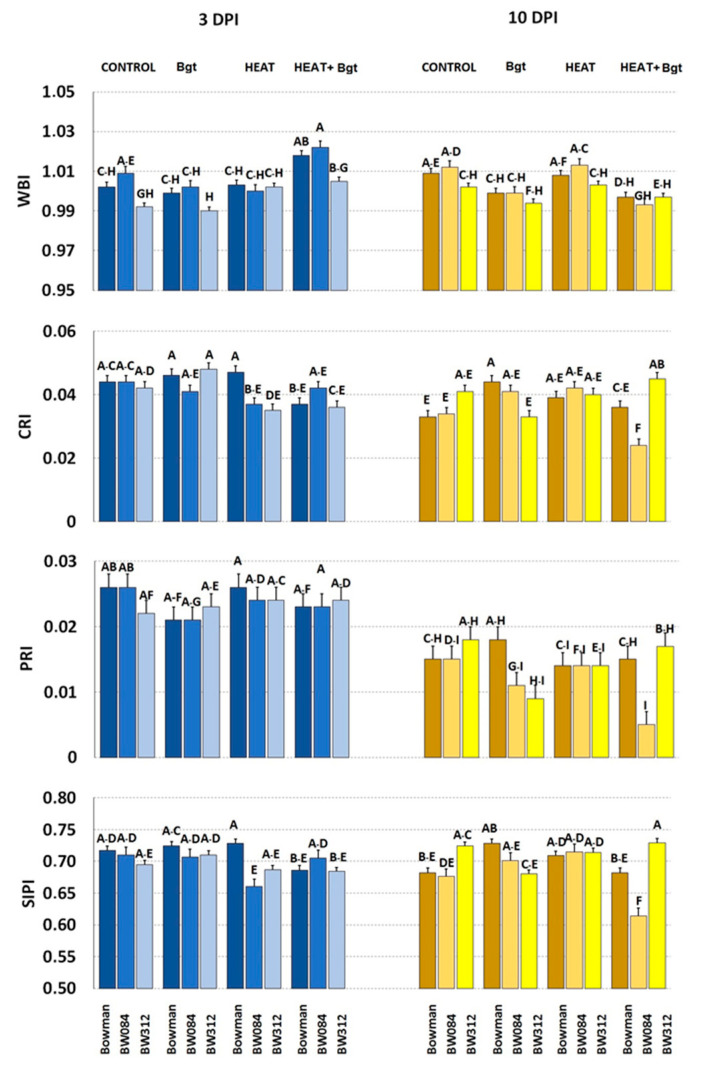
Selected parameters of leaf reflectance characterizing plants of Bowman and its NILs (BW084, BW312) growing under control conditions (Control), inoculated by wheat powdery mildew (*Bgt*), exposed to heat (Heat), and heat pre-treated + inoculated by wheat powdery mildew (Heat+*Bgt*). Mean values (±SD) marked with the same letters did not differ significantly at *p* ≤ 0.05 according to Duncan’s test; DPI—days post inoculation. Heat pre-treated plants were immersed in 49 °C water for 30 s 24 h before inoculation. Measurements were carried out on 11 (3 DPI) and 18 (10 DPI) day old plants.

**Figure 11:**
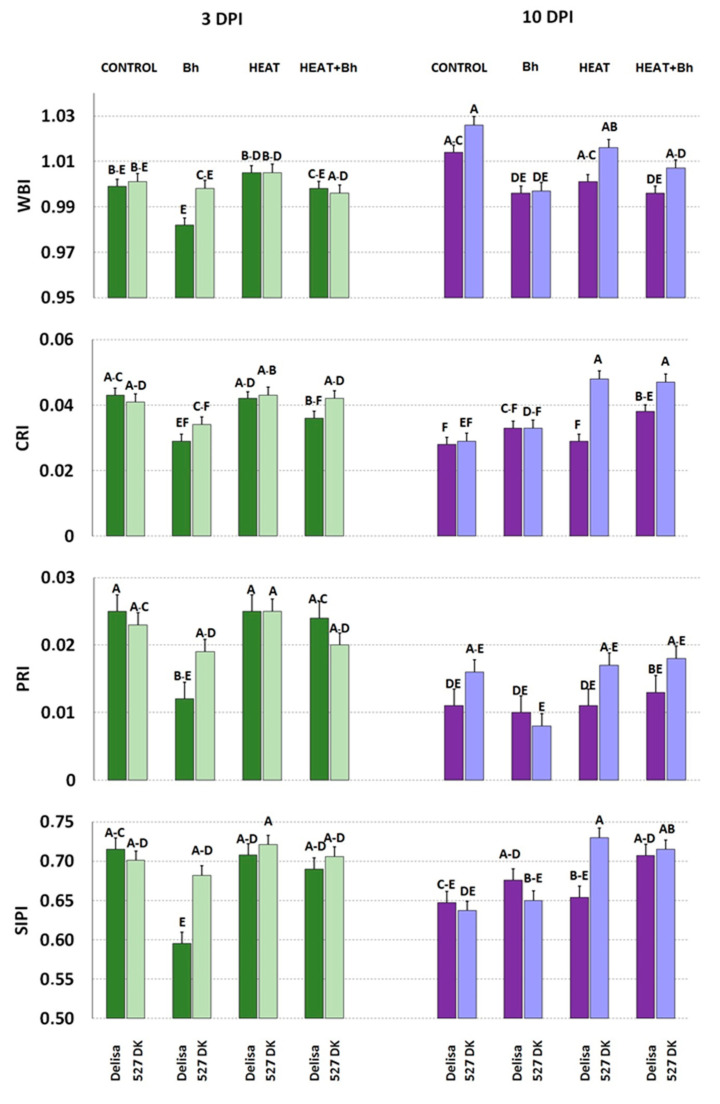
Selected parameters of leaf reflectance characterizing plants of Delisa and its mutant 527DK growing under control conditions (Control), inoculated by barley powdery mildew (*Bh*), exposed to heat (Heat), and heat treated + inoculated by barley powdery mildew (Heat+*Bh*). Mean values marked with the same letters did not differ significantly at *p* ≤ 0.05 according to Duncan’s test; DPI—days post inoculation. Heat pre-treated plants were immersed in 49 °C water for 30 s 24 h before inoculation. Measurements were carried out on 11 (3 DPI) and 18 (10 DPI) day old plants.

Only minor and statistically insignificant changes were observed in any of the measured parameters in the untreated and/or uninoculated barley genotypes as a consequence of mutations (Figure 10 and Figure 11).

At 3 DPI, water content (WBI) increased significantly in wild type and BW 312 only after combined heat treatment and inoculation (Figure 10). Neither heat treatments nor *Bgt* inoculation had an effect on the CRI parameter of Bowman or NILs at 3 DPI. Similarly, Photochemical Reflectance Index (PRI) did not change after heat treatments or *Bgt* inoculation in Bowman or in its NILs. The chlorophyll/carotenoids ratio (SIPI) decreased significantly only in BW084 after heat treatment. 

At 10 DPI, slightly decreased values of WBI were characteristic in plants after *Bh* inoculation, and after heat treatment and *Bh* inoculation (in comparison to control and only heat-treated plants) (Figure 10). Values of CRI were significantly lower only in heat pre-treated and inoculated BW084 plants compared to Bowman. Simultaneously, values of CRI in heat pre-treated and inoculated BW312 were increased when compared to Bowman. In the case of PRI, only combined heat treatment and inoculation decreased its values (and only in BW084). Values of SIPI were significantly lower only in plants of BW084 (heat pre-treated and inoculated) in comparison to Bowman treated in the same way. Meanwhile, SIPI values increased in heat pre-treated and inoculated BW312 when compared to Bowman. 

Regarding the interactions between Delisa and its mutant 527DK with *Bh* race A6 with or without heat treatment at 3 DPI, we did not find significant changes of WBI as a consequence of any of the treatments (Figure 11). Inoculation with barley powdery mildew decreased CRI in Delisa and a similar trend was observed in 527 DK. Furthermore, inoculation with *Bh* decreased PRI and SIPI in Delisa. 

At a later stage (10 DPI) inoculation alone decreased WBI in Delisa and 527 DK (Figure 11). Inoculation alone and heat pre-treatment + inoculation also decreased WBI in Delisa and (statistically insignificantly) in 527 DK. In addition, CRI in 527 DK was increased by heat treatment alone, and by heat + infection in both genotypes. Neither infection nor heat had a significant effect on PRI at this stage in both genotypes. SIPI in 527 DK was elevated by heat treatment, and in both Delisa and 527 DK by combined heat treatment and inoculation.

## 4. Discussion

Climate models predict an increase in average global temperatures of at least 2 °C by the end of the 21st century [48]. It is widely acknowledged that heat stress may increase severity of diseases in species susceptible to fungal pathogens (like powdery mildew) [2,3,49]. Simultaneously, plant species that are resistant to this pathogen might become susceptible [50]. If climate changes intensify, there will be a demand for new genotypes that exhibit resistance to pathogens even in higher temperatures [51].

In preliminary experiments, we found that the interactions between barley powdery mildew race A6 and wild type Bowman as well as some mutant NILs of Bowman (BW084, BW091, BW312, BW333, and BW885) were all compatible, and strong sporulation was observed (data not published). These results were confirmed by our recent observations using two representative genotypes, BW084 (BR-deficient) and BW312 (BR-insensitive). Bowman and its two NILs showed high compatibility to race A6 of barley powdery mildew. Furthermore, Delisa and its mutants 522DK and 527DK were highly incompatible with this pathogen; no visible disease symptoms were detected as we found earlier for 527DK [36]. On the other hand, Bowman and its NILs showed a typical non-host resistance to wheat powdery mildew. It is to be noted that all the above incompatible interactions could be converted to more compatible ones by applying heat pre-treatment, as was observed in the case of barley cultivar Ingrid and its NILs carrying different effective resistance genes against powdery mildew [24]. Thus, we could investigate physiological changes in both compatible and partly incompatible interactions in all genotypes.

By microscopy, we found only one significant difference in the interaction between Bowman and its NILs with barley powdery mildew: the number of non-germinated conidia on Bowman leaves was significantly lower than on BW084 and BW312, indicating a slight increase of resistance in the NILs (Figure 3) and possible role of BR in regulation of this aspect of plant reaction to the pathogen. The microscopic investigations confirmed that the largest number of elongated secondary hyphae was found in the compatible interactions of Bowman and its NILs with barley powdery mildew race A6, or in heat pre-treated Bowman and its NILs infected with wheat powdery mildew. All these results are in correlation with our previous findings that the non-host interaction of seven wheat genotypes with barley powdery mildew resulted in a high number of non-germinated conidia and papillae formation, although to various extents, as compared to the compatible interaction of barley with *Bh* race 6 [21].

In earlier studies of Ali et al. [30] BR signalling mutants of barley were characterized by higher resistance to the necrotrophic net blotch pathogen *Pyrenophora teres* and to *Fusarium culmorum* causing Fusarium head blight, as compared to the wild type plants. According to Goddard et al. [35], the disruption of BRI1 in barley increased disease resistance against necrotrophic and hemibiotrophic pathogens, although it had no effect against biotrophic pathogens. The authors studied NIL pairs of barley two-row Bowman (*HvBRI1*) and Bowman-Uzu (*Hvbri1*), and six-row Akashinriki (*HvBRI1*) and Akashinriki-Uzu (*Hvbri1*).

In host incompatible interactions between Delisa or its mutants and *Bh* race A6, microscopy showed similar patterns as in the case of Bowman or its NILs with *Bgt* in non-host incompatible interactions, except for the higher number of papillae formation and epidermal HR (Figure 3) that indicate a higher level of resistance in the non-host interaction. As with NILs of Bowman, the number of non-germinated conidia was higher on 522DK than on Delisa, but interestingly, 527DK did not show a significant difference in this trait.

Heat pre-treatment markedly induced susceptibility in Delisa and 522DK, but to a much lesser extent in 527DK, indicated by the high number of elongated secondary hyphae and low number of papillae formation on heat-treated Delisa and 522DK, but not on 527DK (Figure 4). The question arises as to why such a strong difference in reaction against powdery mildew disease (especially visible after heat pre-treatment, Figure 1) exists between the two mutants 522DK and 527DK, both characterised by disturbances in BR biosynthesis (missense mutations were identified within the *HvDWARF* [38]). Both mutants have about 60% lower content of brassinosteroid castasterone than Delisa [39]. It appears that in this case, the effect may not be directly connected to BR deficiency, but on the other hand, both mutants carry amino acid substitutions in different functional domains of the encoded enzyme HvDWARF. It should be mentioned that in our previous study, a significant difference in reaction to abiotic stress (elevated temperature) was observed between NILs which carry mutations in different domains of the same protein (BR receptor–HvBRI1) [37]. Irrespective of the causes, the considerably higher resistance of 527DK to barley powdery mildew, maintained even after high temperature stress, seems promising from a practical standpoint for breeding programmes.

Since two NILs with disturbances in BR biosynthesis and perception had slightly higher resistance to powdery mildew (*Bgt*) than their wild type (at 20 °C), and one BR-mutant 527DK had significantly elevated resistance to *Bh* (at 20 °C and after heat stress), one can suppose that BRs play a role in the mechanism responsible for this phenomenon. BR are bound to membrane receptor BRI1, which initiates a phosphorylation cascade and expression of BR-responsive genes [52,53]. One of the first steps in this pathway is binding of complex ligand-receptor (BR/BRI1) to BAK1 (BRI1-associated kinase 1). Simultaneously, BAK1 is also co-receptor to protein FLS2 (receptor for bacterial flagellin peptide (flg22), present in plants) [54]. The flg22/FLS2 complex with BAK1 is involved in initiation of plant defence against bacterial pathogens. Some authors proposed that the BR/BRI1 complex may compete with the flg22/FLS2 complex for BAK1 [55,56]; thus, the elevated level of BR and increased accumulation of BRI1 (by recruiting of BAK1 after bounding) could weaken plant immune response. The opposite situation, namely, a low level of BR or damage of receptor BRI1, could then strengthen the response of plants to pathogens. This model includes a receptor for bacterial peptides, but plant cells also contain receptors capable of recognising fungal pathogens. Receptors containing the lysin motif (LysM) are located on the surface of plant cells and recognize chitin—a polymer of N-acetyl-D-glucosamine, present in fungal cell walls [57,58,59]. Perhaps a similar model as described above for plant-bacteria interaction could be implemented in our studies of plant-fungal interaction, and in this case a lower level of BR or malfunction of BR receptor would be one of the possible explanations for the better resistance of mutant 527DK and NILs to powdery mildew (and other fungal pathogens as described by Ali et al. [30] and Goddard et al. [35] for BR-mutants of barley). Although, according to Wan et al. [58], the chitin signalling pathway (and fungal resistance) mediated by LysM may have a common downstream pathway with the flg22/FLS2 mediated pathways, a clear connection between BR, BAK1 and LysM is presently unknown. On the other hand, according to Albrecht et al. [60], in plants, the mechanism in which BR-mediated growth may antagonize immune signalling also occurs in the case of fungal pathogens, but in a BAK1-independent manner. In *Arabidopsis,* BR pre-treatment inhibited signalling triggered by a fungal PAMP (pathogen-associated molecular pattern), chitin. PAMP is perceived via the LysM-RLK CERK1, and induces PAMP-triggered immunity marker genes in a manner independent of BAK1 [59]. BR pre-treatment inhibited the chitin-induced ROS burst, and the phenomenon also occurred in the null *bak1-4* mutant [60]. 

The role of BR in plant defence systems seems to be complex and can be additionally modified by various factors like abiotic stress (slight elevated resistance of NILs disappeared after heat pre-treatment). But based on our results (together with earlier studies of Ali et al. [30] and Goddard et al. [35]), it can be noted that, at least in some circumstances, a lower content of BR (or disturbed signalling) would be beneficial for plant immune response. Wang [56] points out that BR level and BRI1 accumulation is additionally developmentally regulated–and a lower level of BR is characteristic of less intensively growing older tissues, which may need a modified regulation of resistance mechanisms compared to younger tissues. In case of studied BR-deficient and BR-insensitive barley plants, a lower level of BR and malfunction of receptors are effectively natural traits that in this case can determine the modified response to pathogens–little different then in wild types. In any case, the crucial role of BR as a factor in host and non-host interactions was not entirely confirmed in our work. This is because NILs had only slightly elevated resistance to *Bgt* (in comparison to wild type) and the effect was alleviated by high temperature pre-treatment. On the other hand, in the case of Delisa’s mutants, only 527DK BR-deficient plants were characterised by strong resistance to *Bh* independent of heat shock pre-treatment; the susceptibility of 522DK remains unexplained. However, the significance of BR here (among other factors) should not be excluded.

Since photosynthesis is a very sensitive indicator of stress, we utilized chlorophyll *a* fluorescence to track changes induced by heat and pathogen infection. This method provides information about photosynthetic light reactions mainly connected to PSII.

The O-J phase in the OJIP curve illustrates the state of the PSII donor side and provides information on the antenna size and connectivity between PSII reaction centres [61,62]. The marked increase in fluorescence at step J in Bowman, BW084, and BW312 plants at 10 DPI indicates that the addition of high temperature treatment enhances the negative effect of *Bgt* on limiting electron transport from Q_A_ to Q_B_. The key role of temperature in this process is also emphasized by the clear increase in fluorescence in the O-J and J-I phases, visible in the differential curves in heat-exposed Delisa and heat-exposed 527DK plants. Increasing values in step J and I illustrate the limited number of electron carriers on the acceptor side of PSII [63]. At the same time, the appearance of negative G bands in the I-P phase in Bowman plants and the BW091 and BW312 mutants indicates an increasing number of NADPH molecules per active reaction center under stress conditions [64]. Similar relationships were also observed in the case of other environmental stresses [65,66]. 

Regarding parameters calculated from the chlorophyll *a* fluorescence curves, one can note that due to aging processes in leaves (10 DPI versus 3 DPI), values of parameters like Fv/Fm, PI abs, or ABS/CSm and ETo/CSm, significantly decreased within both cultivars and their mutants (Figure 8 and Figure 9). Considering the performance index, PI abs (parameter synthetically describing PSII efficiency) and comparing untreated and uninoculated (control) Bowman and Delisa plants with their respective mutant control plants, we observed a consistent trend similar to what was previously found in older barley plants growing at a similar temperature of 20 °C [67]. Specifically, BR-deficient and BR-insensitive plants exhibited slightly higher values of PI abs than their wild type plants. 

Summarizing the results of chlorophyll *a* fluorescence measurements, minor changes were recorded in normalized chlorophyll *a* fluorescence induction curves in all genotypes and treatments at 3DPI, in comparison to the untreated control. Disease symptoms were not yet visible at that time, but measurable impairments in photosynthetic light reactions had already become evident, which could have practical value. The effect was further enhanced by 10DPI. Concerning fluorescence parameters, the values of PI abs were statistically significantly lowered already at 3DPI particularly in case of all tested genotypes that underwent heat treatment or were heat pre-treated and inoculated (in comparison to the control). Different reactions (and direction of changes in values of PI abs) were noted only in *Bh*-inoculated plants (Bowman with its NILs).

Values of parameters characterizing leaf spectral properties (based on leaf reflectance) were changed under the influence of stress factors in most of cases, though not substantially. However, regarding changes of relative water status (WBI parameter), data obtained in this experiment are in agreement with our earlier findings [36,68]. Consequently, after inoculating barley with powdery mildew, there was a noticeable decrease in water content in leaves, typically observed between 7 DPI to 10 DPI. This trend was observed not only in powdery mildew susceptible genotypes, but also in resistant genotypes such as NILs of Ingrid or highly resistant line 527DK. This may suggest that the decrease in water content could be attributed to micro-injuries caused by the presence of pathogens on the leaf surface. Interestingly, in case of 527DK the effect of water loss after inoculation was modified by exposure to heat stress. The plants exposed to heat or to a combination of heat pre-treatment and inoculation maintain high WBI values (very similar to untreated control).

Regarding the other reflectance parameters (CRI, SIPI), consistency with earlier results [36,68] is lower, indicating that other factors (like soil composition) might be important. As mentioned above, probably due to aging processes in plants, values of chlorophyll *a* parameters such as Fv/Fm or PI abs were reduced in all tested genotypes (10 DPI). Leaf reflectance measurements allowed for the calculation of the Photochemical Reflectance Index (PRI), which also serves as a simplified indicator of photosynthetic efficiency. Values of PRI were generally lower at 10 DPI than at 3 DPI for all genotypes tested, somewhat corresponding with results of chlorophyll *a* fluorescence.

## 5. Conclusions

Selected cultivars were chosen to study the role of brassinosteroids in host and non-host interactions of barley with powdery mildew, presenting an intriguing model. Specifically, Bowman and its NILs with disturbances in biosynthesis/signaling of BR showed high compatibility to barley powdery mildew race A6 (host plant—pathogen interaction). In contrast, cultivar Delisa and its BR-deficient mutants 522DK and 527DK were entirely incompatible with this pathogen. On the other hand, Bowman and its NILs exhibited complete resistance to wheat powdery mildew, representing a non-host plant-pathogen interaction. Heat pre-treatment generally converted these incompatible interactions into more compatible ones. The crucial role of BR as factors in host and non-host interactions was not entirely confirmed, although the significance of these hormones (among other factors) cannot be excluded. Two observations may support this notion: (1) in barley powdery mildew-inoculated plants there was a significant difference between wild type Bowman and its NILs; the number of non-germinated conidia significantly increased on both NILs indicating a certain elevation of resistance in NILs, and (2) mutant 527DK displayed much weaker visible symptoms upon barley powdery mildew infection even after heat pre-treatment, suggesting its potential as an intriguing candidate for breeding programs. If climate changes intensify, there will be a demand for new genotypes that exhibit resistance to pathogens even in higher temperatures.

## Data Availability

Data are contained within the article and Supplementary Materials.

## Supplementary Materials

The following supporting information can be downloaded at: https://www.mdpi.com/article/10.3390/life14010160/s1. Table S1. Selected parameters of chlorophyll *a* fluorescence characterizing PSII efficiency in plants of Bowman and its NILs (BW084, BW312) growing under control conditions (Control), inoculated by wheat powdery mildew (*Bgt*), exposed to heat (Heat), and heat pre-treated + inoculated by wheat powdery mildew (Heat+*Bgt*). Mean values marked with the same letters did not differ significantly at *p* ≤ 0.05 according to Duncan’s test; DPI–days post inoculation. Heat pre-treated plants were immersed into 49 °C water for 30 s 24 h before inoculation. Measurements were carried out on 11 (3 DPI) and 18 (10 DPI) day old plants. Table S2. Selected parameters of chlorophyll *a* fluorescence characterizing PSII efficiency in plants of Delisa and its mutant 527DK growing under control conditions (Control), inoculated by barley powdery mildew (*Bh*), exposed to heat (Heat), and heat pre-treated + inoculated by barley powdery mildew (Heat+*Bh*). Mean values marked with the same letters did not differ significantly at *p* ≤ 0.05 according to Duncan’s test; DPI–days post inoculation. Heat pre-treated plants were immersed into 49 °C water for 30 s 24 h before inoculation. Measurements were carried out on 11 (3 DPI) and 18 (10 DPI) day old plants.
