# Supplementary material for: Heat Pre-Treatment Modified Host and Non-Host Interactions of Powdery Mildew with Barley Brassinosteroid Mutants and Wild Types
Source: Life (Basel). 2024 Jan 22;14(1):160. doi: 10.3390/life14010160 (PMC10817351; doi:10.3390/life14010160)
Supplement: Supplementary file 1 [file life-14-00160-s001.zip › life-2813600-supplementary.pdf]

Supplementary data:

Table S1. Selected parameters of chlorophyll *a* fluorescence characterizing PSII efficiency in plants of Bowman and its NILs (BW084, BW312) growing under control conditions (Control), inoculated by wheat powdery mildew (*Bgt*), exposed to heat (Heat) and heat pre-treated + inoculated by wheat powdery mildew (Heat+*Bgt*). *Mean values marked with the same letters did not differ significantly at p ≤ 0.05 according to Duncan's test; DPI – days post inoculation.* Heat pre-treated plants were immersed into 49 °C water for 30 s 24 h before inoculation. Measurements were carried out on 11 (3DPI) and 18 (10 DPI) days old plants.

Parameters	Treatment	3 DPI			10 DPI		
		Bowman	BW084	BW312	Bowman	BW084	BW312
Fv/F_0	Control	4.683^{A}	4.730^{A}	4.675^{A}	3.845^{CD}	3.308^{F}	3.770^{DE}
	Bgh	4.703^{A}	4.702^{A}	4.730^{A}	3.612^{E}	3.332^{F}	3.022^{G}
	Heat	4.452^{B}	4.549^{AB}	4.569^{AB}	4.001^{C}	3.914^{CD}	3.920^{CD}
	Heat+*Bgt*	4.642^{A}	4.670^{A}	4.400^{B}	2.970^{G}	3.266^{F}	3.026^{G}
ABS/RC	Control	1.950^{K}	1.975^{K}	1.993^{K}	2.308^{EFG}	2.479^{CD}	2.366^{EF}
	Bgt	1.948^{K}	1.962^{K}	2.020^{JK}	2.376^{EF}	2.400^{DF}	2.541^{BC}
	Heat	2.042^{JK}	2.094^{IJ}	2.103^{IJ}	2.239^{GH}	2.275^{FG}	2.326^{EFG}
	Heat+*Bgt*	1.951^{K}	1.984^{K}	2.173^{HI}	2.554^{BC}	2.597^{B}	2.773^{A}
DIo/RC	Control	0.346^{H}	0.345^{H}	0.342^{H}	0.477^{EF}	0.568^{C}	0.486^{E}
	Bgt	0.342^{H}	0.343^{H}	0.353^{H}	0.526^{D}	0.547^{CD}	0.618^{B}
	Heat	0.378^{GH}	0.378^{GH}	0.375^{GH}	0.448^{F}	0.463^{EF}	0.477^{EF}
	Heat+*Bgt*	0.347^{H}	0.352^{H}	0.405^{G}	0.615^{B}	0.617^{B}	0.703^{A}
TRo/RC	Control	1.606^{I}	1.631^{I}	1.635^{I}	1.831^{EF}	1.924^{C}	1.858^{CDE}
	Bgt	1.605^{I}	1.607^{I}	1.668^{HI}	1.862^{CDE}	1.846^{DE}	1.909^{CD}
	Heat	1.659^{HI}	1.712^{GH}	1.736^{G}	1.815^{EF}	1.811^{EF}	1.854^{DE}
	Heat+*Bgt*	1.611^{I}	1.636^{I}	1.768^{FG}	2.006^{B}	1.986^{B}	2.079^{A}
ETo/RC	Control	0.928^{FGHI}	0.949^{CDEFG}	0.968^{BCDE}	0.924^{GHIJ}	1.034^{A}	0.970^{BCDE}
	Bgt	0.960^{BCDEF}	0.943^{EFGH}	0.975^{BC}	0.942^{DEFG}	0.979^{B}	1.011^{A}
	Heat	0.942^{EFG}	0.973^{BCD}	0.978^{B}	0.886^{KL}	0.888^{KL}	0.913^{HIJK}
	Heat+*Bgt*	0.896^{JKL}	0.918^{GHIJ}	0.982^{B}	0.889^{KL}	0.877^{L}	0.915^{IJK}
$\varphi_{(Eo)}$	Control	0.579^{BCDE}	0.582^{ABCD}	0.588^{ABC}	0.505^{JK}	0.539^{H}	0.522^{I}
	Bgt	0.596^{A}	0.590^{AB}	0.585^{ABC}	0.507^{J}	0.531^{HI}	0.538^{H}
	Heat	0.569^{DEFG}	0.568^{EFG}	0.575^{CDEF}	0.494^{JKL}	0.491^{KL}	0.487^{L}
	Heat+*Bgt*	0.562^{FG}	0.561^{G}	0.556^{G}	0.445^{M}	0.442^{M}	0.441^{M}
DIo/CSm	Control	318^{KL}	331^{IJKL}	338^{HIJK}	360^{DEFF}	376^{CD}	358^{EFG}
	Bgt	326^{JKL}	315^{L}	317^{L}	374^{C}	368^{CDE}	378^{C}
	Heat	341^{GHIJ}	328^{JKL}	336^{HIJK}	359^{EFG}	353^{EFGH}	356^{EFG}
	Heat+*Bgt*	348^{FGHI}	328^{JKL}	351^{EFGH}	425^{AB}	423^{B}	440^{A}
TRo/CSm	Control	1493^{B}	1532^{B}	1480^{B}	1387^{CDE}	1244^{G}	1324^{F}
	Bgt	1538^{B}	1476^{B}	1498^{B}	1376^{CDEF}	1241^{G}	1207^{G}
	Heat	1504^{B}	1503^{B}	1526^{B}	1428^{C}	1402^{CD}	1366^{DEF}
	Heat+*Bgt*	1612^{A}	1533^{B}	1536^{B}	1361^{DEF}	1338^{EF}	1352^{DEF}

Table S2. Selected parameters of chlorophyll *a* fluorescence characterizing PSII efficiency in plants of Delisa and its mutant 527DK growing under control conditions (Control), inoculated by barley powdery mildew (*Bh*), exposed to heat (Heat) and heat pre-treated + inoculated by barley powdery mildew (Heat+*Bh*). *Mean values marked with the same letters did not differ significantly at p ≤ 0.05 according to Duncan's test; DPI – days post inoculation.* Heat pre-treated plants were immersed into 49 °C water for 30 s 24 h before inoculation. Measurements were carried out on 11 (3DPI) and 18 (10 DPI) days old plants.

Parameters	Treatment	3 DPI		10 DPI	
		Delisa	527DK	Delisa	527DK
F_v/F_0	Control	4.444^{BC}	4.681^{A}	3.066^{F}	3.303^{F}
	Bh	4.412^{BCD}	4.513^{ABC}	3.692^{E}	3.822^{E}
	Heat	4.490^{ABC}	4.571^{AB}	3.699^{E}	3.676^{E}
	Heat+*Bh*	4.275^{CD}	4.174^{D}	3.690^{E}	3.689^{E}
ABS/RC	Control	2.030^{EFGH}	2.000^{H}	2.393^{A}	2.369^{A}
	Bh	2.094^{DEFGH}	2.059^{FGH}	2.194^{BCD}	2.172^{BCD}
	Heat	2.133^{DEFG}	2.020^{GH}	2.312^{AB}	2.311^{AB}
	Heat+*Bh*	2.208^{CDEF}	2.200^{BCDE}	2.255^{ABC}	2.242^{ABC}
DIo/RC	Control	0.380^{DE}	0.359^{E}	0.566^{A}	0.569^{A}
	Bh	0.388^{CDE}	0.374^{DE}	0.463^{B}	0.452^{B}
	Heat	0.388^{CDE}	0.363^{E}	0.488^{B}	0.488^{B}
	Heat+*Bh*	0.422^{CD}	0.427^{C}	0.482^{B}	0.480^{B}
TRo/RC	Control	1.657^{EF}	1.645^{F}	1.801^{AB}	1.855^{A}
	Bh	1.706^{CDEF}	1.685^{DEF}	1.725^{BCDE}	1.720^{BCDE}
	Heat	1.733^{BCDEF}	1.657^{EF}	1.824^{AB}	1.823^{AB}
	Heat+*Bh*	1.786^{BCD}	1.774^{ABCD}	1.773^{ABC}	1.762^{ABC}
ETo/RC	Control	1.006^{ABC}	0.972^{CDEF}	1.025^{A}	1.009^{ABC}
	Bh	0.969^{BCDE}	0.936^{FG}	0.934^{FG}	0.942^{EFG}
	Heat	0.974^{BCDEF}	0.951^{DEFG}	0.928^{G}	0.857^{H}
	Heat+*Bh*	0.994^{BCD}	1.021^{AB}	0.978^{BCDE}	0.944^{EFG}
$\varphi_{(Eo)}$	Control	0.599^{A}	0.589^{A}	0.565^{BC}	0.545^{DEF}
	Bh	0.569^{B}	0.560^{BCD}	0.538^{EF}	0.539^{DEF}
	Heat	0.574^{B}	0.562^{BC}	0.509^{G}	0.477^{H}
	Heat+*Bh*	0.558^{BCD}	0.572^{B}	0.553^{CDE}	0.530^{F}
DIo/CSm	Control	318^{EFG}	304^{G}	355^{ABC}	340^{ABCDE}
	Bh	309^{FG}	314^{FG}	353^{ABC}	336^{BCDEF}
	Heat	344^{ABCD}	322^{DEFG}	363^{A}	328^{CDEFG}
	Heat+*Bh*	336^{BCDEF}	326^{CDEFG}	357^{AB}	312^{EFG}
TRo/CSm	Control	1416^{BCD}	1444^{BC}	1094^{HI}	1085^{I}
	Bh	1377^{BCDE}	1419^{BCD}	1299^{FG}	1279^{G}
	Heat	1531^{A}	1461^{AB}	1360^{DEF}	1245^{G}
